# History and definitions of AI

**DOI:** 10.1192/j.eurpsy.2025.238

**Published:** 2025-08-26

**Authors:** E. Chumakov

**Affiliations:** Department of Psychiatry and Addiction, St Petersburg State University, St Petersburg, Russian Federation

## Abstract

**Abstract:**

Artificial Intelligence (AI) has evolved from philosophical musings to a crucial component of modern technology. The concept of using computer technology to simulate thinking and intelligent behavior was first described by Alan Turing in 1950. The Dartmouth Conference in 1956 marked a pivotal moment, coining the term **“**artificial intelligence**”** and fostering interdisciplinary collaboration. Further contributions to the development of AI theory were made by scholars such as John McCarthy, Valerie Barr, Edward Feigenbaum, Claude Shannon and others. The concept of creating AI is to form a system that would be able to operate autonomously, solving intellectual tasks in a manner similar to human cognitive processes. This is achieved by using algorithms that are translated into computer code containing instructions for quickly analyzing and transforming data into conclusions, information, or other outputs. Throughout the subsequent decades, AI experienced cycles of optimism and disillusionment, known as **“**AI winters, **”** primarily due to unmet expectations regarding computational capabilities and practical applications. The resurgence of AI in the 21st century can be attributed to breakthroughs in machine learning, especially deep learning, and the availability of vast datasets and increased computational power. Today, AI is defined broadly as the capability of a machine to imitate intelligent human behavior, encompassing a range of subfields including natural language processing, robotics, and computer vision. As AI continues to evolve, its definitions are adapted to address ethical considerations and the implications of autonomy in decision-making systems. Current discourse often examines AI not only as a technological phenomenon but also as a sociocultural force, raising questions about accountability, bias, and the future of work. This presentation will reflect the complex path of AI development and the shifting paradigms that define its scope and impact on modern society.

**Disclosure of Interest:**

None Declared

